# The role of ERG current in pacemaking and bursting in dopamine neurons

**DOI:** 10.1186/1471-2202-12-S1-P27

**Published:** 2011-07-18

**Authors:** Marco A Huertas, Huifang Ji, Kristal Tucker, Edwin Levitan, Paul D Shepard, Carmen C Canavier

**Affiliations:** 1 Neuroscience Center of Excellence, LSU Health Sciences Center, New Orleans, LA 70112, USA; 2 Department of Pharmacology and Chemical Biology, University of Pittsburgh, Pittsburgh, PA 15261, USA; 3 Maryland Psychiatric Research Center, University of Maryland School of Medicine, Baltimore, MD 21201, USA

## 

Dopamine (DA) neurons exhibit regular, pacemaking firing in the range of 1-8 Hz in *in vitro* preparations and are observed to produce bursts of action potentials at much higher frequencies in *in vivo* studies. The model used in the present study expands on our previously published compartmental DA neuron model [1] and incorporates improved descriptions for the L-type Ca^2+^[2], Na [3] and ERG currents [4]. The approach is to calibrate the model using various types of data acquired *in vitro* in order to confidently model and explain the effects of drug application *in vivo*. One validation of the new model dynamics is its ability, without further calibration, to simulate a novel type of bursting observed *in vitro* when the SK current is blocked. In this type of bursting, the silent phase of the burst is a depolarized plateau, and the active phase consists of spiking with increasing frequency leading into a brief but intense burst of spike terminating in depolarization block [5].

Here we focus on the role of the Ether-a-go-go-related gene (ERG) K^+^ current. The ERG current has the unusual characteristic that channels inactivate faster than they activate, and upon recovery from inactivation they pass through the open state before they enter the closed state. Our model predicts that enough of these channels remain in the open state during pacemaking to slow the frequency of pacemaking, even though there is not sufficient activation of these channels at hyperpolarized potentials to affect the input resistance. Indeed, we have shown that blocking the ERG current in a slice preparation with either E4031 or the toxin rBeKm-1 dose-dependently increases the spontaneous firing rate by 50% without significantly altering the membrane resistance. The model includes background noise levels and can faithfully reproduce the increased precision of DA cell firing when the ERG current is blocked. Furthermore, we have conducted dynamic clamp experiments in which bursts are evoked by injecting a square pulse of virtual NMDA conductance in the soma. Blocking the ERG current shortens the burst because the spike frequency under these conditions is also increased. As a result, the burst is terminated as the cell enters into depolarization block sooner than in the absence of this current. In contrast, local application of E-4031 via passive diffusion from an extracellular recording electrode *in vivo* caused a decline in the number of spike doublets observed and a concomitant increase in bursts comprised of 3 to 5 spikes per burst. The difference in the effect of ERG block under these two conditions is likely due to the difference between evoking a single burst using the dynamic clamp, and the burst dynamics *in vivo* that are regulated by the interactions between the L-type Ca^2+^, NMDA, SK current and ERG currents. The model also predicts a role for the ERG current in termination of depolarized plateaus [6] that follow spike bursts.
